# Criticality Hidden in Acoustic Emissions and in Changing Electrical Resistance during Fracture of Rocks and Cement-Based Materials

**DOI:** 10.3390/ma13245608

**Published:** 2020-12-09

**Authors:** Gianni Niccolini, Stelios M. Potirakis, Giuseppe Lacidogna, Oscar Borla

**Affiliations:** 1 Department of Structural, Geotechnical and Building Engineering, Politecnico di Torino, C.so Duca degli Abruzzi 24, 10129 Torino, Italy; gianni.niccolini@polito.it (G.N.); oscar.borla@polito.it (O.B.); 2 Department of Electrical and Electronics Engineering, University of West Attica, 12244 Egaleo, GR-12244 Athens, Greece; spoti@uniwa.gr

**Keywords:** acoustic emission, electrical resistance, damage monitoring, criticality, natural time analysis

## Abstract

Acoustic emissions (AE) due to microcracking in solid materials permit the monitoring of fracture processes and the study of failure dynamics. As an alternative method of integrity assessment, measurements of electrical resistance can be used as well. In the literature, however, many studies connect the notion of criticality with AE originating from the fracture, but not with the changes in the electrical properties of materials. In order to further investigate the possible critical behavior of fracture processes in rocks and cement-based materials, we apply natural time (NT) analysis to the time series of AE and resistance measurements, recorded during fracture experiments on cement mortar (CM) and Luserna stone (LS) specimens. The NT analysis indicates that criticality in terms of electrical resistance changes systematically precedes AE criticality for all investigated specimens. The observed greater unpredictability of the CM fracture behavior with respect to LS could be ascribed to the different degree of material homogeneity, since LS (heterogeneous material) expectedly offers more abundant and more easily identifiable fracture precursors than CM (homogenous material). Non-uniqueness of the critical point by varying the detection threshold of cracking events is apparently due to finite size effects which introduce deviations from the self-similarity.

## 1. Introduction

A crucial question of scientists and civil engineers concerns the use of the electrical properties of geological and engineering materials as potential precursors of structural collapses and earthquakes [1,2,3,4,5,6,7,8,9]. The behavior of electrical properties has been used for several years in induced polarization, resistivity and electromagnetic methods in the context of likely mechanisms, for both piezoelectric and non-piezoelectric materials [10,11]. There are many experimental techniques available for rocks, ionic crystals and concrete-like materials, including the observation of changes in electrical properties, e.g., electrical resistance or resistivity [12,13,14,15,16], and of electrical and electromagnetic signals as functions of the applied external load. A combination of techniques involves also the observation of electrical properties as functions of applied electromagnetic (EM) fields (to test for damage-induced voltage-current non-linearity) and of EM frequency, including variations in relevant environmental parameters (temperature, water content, etc.) [17,18].

During mechanical loading of materials, fracture-induced electrical currents, acoustic emissions (AE) and electromagnetic emissions (EME) allow the real-time monitoring of damage evolution [8,9,10,11,12,19,20]. While the origin of AE from materials experiencing damage is well understood [21,22], different models have been proposed to explain the genesis of electrical and electromagnetic signals related to irreversible phenomena, such as the formation of electrical charges due to the breakage of bonds [23], the discharge model [24], the capacitor model [25], the surface oscillation model [26] and the moving charge dislocation model [27]. During compression tests, stress-induced polarization currents—attributed either to the well-known piezoelectric effect for polycrystalline natural rocks with piezoelectric properties (granite, quartzite), or to the moving segments of charged edge dislocations for non-piezoelectric ionic crystals (LiF) [10,11]—are detected in the sample. In complex materials (granite) containing quartz inclusions, AE originating from the microfracturing process stimulate damping vibrations of the quartz grains—polarized due to the high stress levels—which act as sources of EME. As far as non-piezoelectric materials (pure ionic crystals) are concerned, the motion of segments of charged dislocations—piling up during crack initiation and propagation—with respect their compensating point defects are held responsible for generating EME.

Recent accumulated laboratory evidence indicates that the generation of freshly formed fracture surfaces, due to opening cracks, is accompanied by simultaneous EME and AE, whereas AE signals that are not associated with EME signals are due to frictional noises during the slip between pre-formed fracture surfaces [28,29]. For this reason, EME is being increasingly considered as a precursory signal, since it is argued that it is produced only during the generation of new fresh surfaces/rupture of bonds, due to cracking in the material. In this regard, it has been observed that the larger the stress drops, the more intense the EME activity [30].

Furthermore, it should be stressed the compatibility of recently performed laboratory fracture experiments on rocks and ionic crystals with the processes occurring in the Earth’s crust during the earthquake preparatory stage. The experimental evidence reveals that the final stage of the failure process coincided in time with the maximum of AE and quiescence in EME, while strong avalanche-like EME precedes this phase. Then, an EME silence is observed just before the final collapse in the laboratory, as well as at the geophysical scale before the seismic shock [8,9,10,11].

Stress-induced currents and EME are detected also from cement-based materials under compressive loading, where electrical double layer formation and motion of ions have been proposed as the possible causes of the observed EME: layers of ions in the water inside the capillary pores accelerate —as the pore solution moves upon loading—with respect to oppositely charged layers in the solid region, resulting in a time-varying dipole moment which generates EME [31,32,33,34]. Since the electrical conduction of rocks and mortar is largely electrolytic [35], the electrical resistance in relatively dry materials should reasonably increase as a result of microcracking, which breaks the existing conductive network within the material. Since growing microcracks are AE sources, a correlation between electrical resistance changes and AE bursts are eventually expected.

The present goal is to investigate the correlation between electrical resistance and AE measurements, carried out in air-dry surface cement mortar and rock specimens subjected to fracture tests. Here, the application of the well-known AE technique aims to verify the reliability of the electrical resistance measurement, which would enable damage monitoring with simple and inexpensive equipment.

In order to further investigate the possible critical behavior of fracture processes through their observables, the AE and the electrical resistance, we proceed here to the analysis of AE and electrical resistance time series using first classical definitions of damage in Kachanov’s sense [36] and then natural time (NT) analysis [37,38,39], a recently proposed method in the framework of critical phenomena [8,40,41,42,43]. The following sections describe the experimental setup and the fracture experiments performed, as well as damage measurements based on the acquired AE and electrical signals; then, a brief description of the key concepts and basic formulas of the NT analysis method; after that, the NT analysis of the AE and electrical resistance signals, and finally a summary of the main findings.

## 2. Experimental Setup—AE Signals and Electrical Resistance Changes

**Figure 1:**
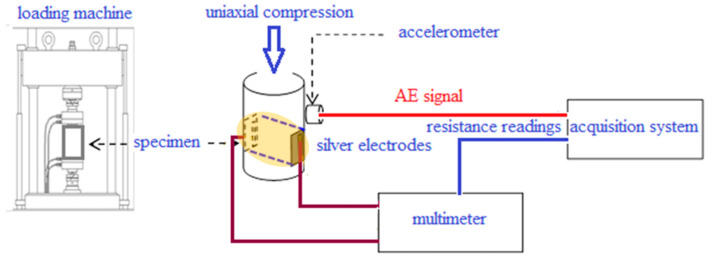
Experimental setup (the yellow region between the electrodes, marked by a dotted outline, defines the volume through which most of electric charges are expected to flow). Adapted from [44].

**Figure 2:**
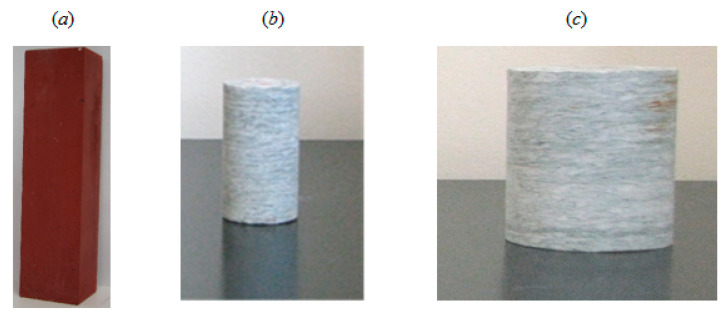
Blocks of cement mortar (**a**) and rods of Luserna stone (**b**,**c**).

A schematic diagram of the experimental setup and pictures of the test specimens are shown in Figure 1 and Figure 2. The experiments were carried out on three rods of Luserna stone (with fixed height, 50 mm, and variable diameter, 52 mm for two specimens and 25 mm for the remaining one), and two blocks of cement mortar (section 40 × 40 mm^2^, height 160 mm) enriched with ferric oxide to improve the electrical conductivity. Using different shapes (cylindrical vs. prismatic) necessitates some corrections to make the results comparable, as the post-peak behavior of prisms under compression is more ductile than that of cylinders. Such shape effects were avoided by making cement mortar specimens more slender (slenderness equal to 160/40 = 4) in order to induce a brittle collapse once the peak stress was reached.

Since mortar (as well as Luserna stone) has a high content of electrical insulator (silicon dioxide with electrical resistivity equal to 10^14^ Ω cm), an increase of 10% of ferric oxide (electrical resistivity equal to 10^9^ Ω cm) enhances its electrical conductivity. The chemical composition of both materials is reported in Table 1.

Both specimens were put under uniaxial compression till macroscopic fracture, using a 500 kN servo-hydraulic loading machine equipped with electronic control in order to conduct tests at constant displacement rate (1 μm s^–1^ applied to Luserna stone and 2 μm s^–1^ to cement mortar). Such low rates were used to induce a relatively more ductile response from the specimen, as being characterized by a number of AE signals—suitable for statistical analysis—greater than that generally observed at higher loading (or strain) rates. The electrical resistance of the specimens was measured by the two-electrode technique, using an Agilent 34411 A multimeter capable of measuring resistances as high as 1 GΩ. After placing a constant voltage *V* in series with the multimeter and the unknown resistance, the current flowing through the specimen was measured, thus yielding the resistance from the Ohm’s law. Brass screws and copper wires served as electrical contacts, placed on opposite faces of the specimen where a 30 × 30 mm^2^ area was coated with a conducting silver paint in order to minimize the contact resistance (see Figure 1, and [44] for further details).

The electrical resistance *R*_0_ of each virgin specimen (shown in Figure 2a–c) was measured at the beginning of the test. Then, the electrical resistance *R* of the damaged specimen was measured up to fracture, at a sampling rate of 25 Hz. The reported values were obtained by averaging over 100 samples and expressed in terms of *R*/*R*_0_.

Acoustic emissions were measured by a calibrated accelerometer (charge sensitivity 9.20 pC/m s^–2^) at a frequency range from a few hertz to 10 kHz to detect low-frequency signals generated by larger fractures, generally occurring as the failure is approaching [45,46] and held responsible for the breaking of material’s conductive network. Previous experimental campaigns with transducers working in different frequency ranges (0.1–10 vs. 50–500 kHz) demonstrated a systematic reduction of the AE signal frequencies over damage accumulation [45].

AE signals were transmitted from the accelerometer to a 20 dB low-noise amplifier and then acquired at the audio sampling rate of 44.1 kHz by a sound card. Each AE signal was recorded through a hit-based method, where the HDT value was 68.1 μs (i.e., three times the delay 22.7 μs between the consecutive execution of sample taking): the end of the signal was recognized when the sum of signal readings per 68.1 μs or more descended under the threshold level. The HLT value was 68.1 μs (to avoid repeated recognitions of the same signal) and PDT was 45.4 μs. 

**Figure 3:**
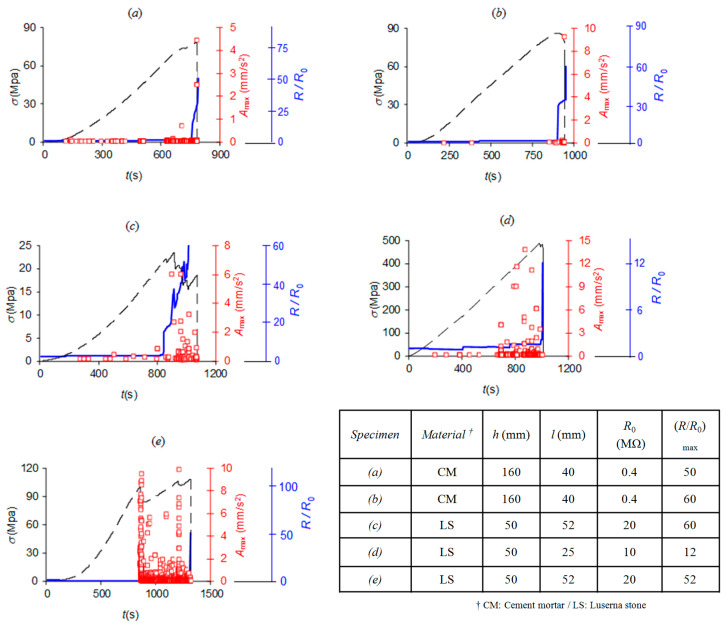
Variations over time of the applied stress (black dashed line), electrical resistance in *R*_0_ units (blue solid line) and AE activity (red squares representing the peak acceleration of each AE event). The insert table provides information about the specimens corresponding to the data presented in (**a**–**e**).

The AE signals were characterized by the time of occurrence and the magnitude, expressed as *A*_dB_ = 20 log_10_ (*A*_max_/ 1 μm s^–2^), where *A*_max_ is the peak acceleration on the specimen surface produced by the AE wave [47]. Signal attenuation effects were not considered relevant because of the small distances involved (even if such effects, including scattering, become increasingly relevant during the damage process). The detection threshold was set to 40 dB in order to filter electrical disturbances and noisy signals, whereas a post-process FFT signal analysis was used to identify and filter the mechanical noise of the loading machine. Therefore, the AE time series, the accumulated number of AE events, the load history and the relative resistance *R*/*R*_0_ are plotted in Figure 3.

Load vs. time diagrams of Figure 3a,b illustrate a brittle response by both mortar specimens. Despite the absence of relevant deviations from the linear elastic behavior, the increase in electrical resistance and AE activity revealed progressive damage accumulation within the specimen.

Except for one case depicted in Figure 3d, the load vs. time diagrams of the Luserna stone specimens were, on the contrary, characterized by a more ductile behavior, with numerous intermediate load drops (see Figure 3c,e) correlated to clusters of AE events and changes in electrical resistance, all signs revealing the preparatory damage stage of the specimen failure.

The different mechanical and electrical behaviors of the two tested materials were ascribed to their physical-mechanical properties: the Luserna stone has a heterogeneous and porous crystalline structure, whereas the cementitious specimen is an artificial material, compacted during the manufacturing process, and thus more homogeneous.

## 3. Damage Measurements Based on AE and Electrical Resistance Time Series

The phenomenon of damage in rocks and concrete-like materials often consists of surface discontinuities in the form of inner microcracks (e.g., decohesions of interfaces between cement, sand and aggregates in concrete), or of volume discontinuities in the form of voids (responsible for measurable macroscopic volume changes). In the sense of continuum mechanics, such a discontinuous state is represented by a continuous damage variable *D*, representing the surface density of microcracks and cavities in any plane of a representative volume element *V*, i.e., the smallest volume on which a mean value of a defect characteristic may represent a field of discontinuous properties. If S is a cross-sectional area (with normal **n**) of *V*, with a total area $\bar{S}$ of the defect traces, $D_{n}=\bar{S}\;/S$ represents the local damage with respect to the **n**-direction. Considering only isotropic damage, i.e., in the case of defects without preferred orientation, damage is completely characterized by a scalar variable: $D_{n}=D$, ∀**n** for each volume element, although the accuracy of isotropic damage models generally decreases since material symmetries change during the loading process For rocks and concrete-like materials, the linear size of the representative volume element is of the order of 10.0 to 100.0 mm, which is that of the test specimens. Therefore, the evolving damage state of the current specimens is representable by a scalar function [48,49,50].

A full set of methods of non-direct damage measurement have been developed through its effects on strain properties. It is possible to consider the variable *D* as an internal variable in the sense of the thermodynamics of irreversible processes, where energy is released or dissipated in the damage processes of the creation of a new discontinuity (fracture) surface.

Considering a freshly formed crack of area S as a source of acoustic emissions, the accumulated damage can be expressed as a sum of acoustic emissions:

(1) $$D\propto \sum_{i}10^{m_{i}}$$

where *m = A*_dB_/20 is the magnitude of an AE event, and it is proportional also to the logarithm of the source crack area *S*. The following relationships have been established [47]:

(2) $$m\propto \frac{2}{3}c\;\log_{10}A_{\max}\propto \;\log_{10}S$$

where the factor c depends on the type of transducer: c=1 if the sensor acts as a strain-meter, c=1.5 for a velocity transducer, and c=3 in the case of an accelerometer. Here, being c=3, the accumulated state of damage in terms of released energy by AE is given by the sum of the squared AE peak amplitudes, $D\propto \sum_{i}A_{\max,i}{}^{2}$. The cumulative damage is normalized to one, where D=1 is the maximum damage at the moment of failure.

In recent years, the correlation of electrical resistance with damage in solids has been investigated as well. As the failure is approached, the opening of micro- and macrocracks produces more void space in the material and, consequently, higher electrical resistance.

Considering the undamaged specimen before loading, the electrical resistance $R_{0}$ between the electrodes is given in terms of resistivity *ρ* by:

(3) $$R_{0}=\rho l/S$$

where *l* is the distance between the electrodes, i.e., the distance between opposite faces of the specimen, and *S* is the cross-sectional area of the cylindroid defined by the electrodes’ surfaces (see the dashed blue contour in Figure 1) and through which, roughly, electrical charges flow.

Since an accurate calculation of the electrical resistance of the damaged specimen is extremely difficult, some simplifying assumptions appear to be necessary.

The electrical resistance of the considered volume, which experiences damage during the test, can be expressed as:

(4) $$R=\frac{\rho^{\prime }l^{\prime }}{S^{\prime }}=\frac{\rho l}{S\left(1-D\right)}$$

where changes in length and resistivity (the latter due to changes in porosity) are neglected, $l^{\prime }=l$ and $\rho^{\prime }=\rho$, the damage is assumed to be uniformly distributed along the axial length l and the cross-section S of the cylindroid. In this simplified model, changes in electrical resistance are entirely attributed to changes in the effective current-conducting area of the cylindroid, which is identified by its undamaged cross-sectional area, $S^{\prime }=\left(1-D\right)S$. Thus, combining Equations (3) and (4) gives the simplest definition of the damage variable based on electrical resistance changes [46]:

(5) $$D=1-R_{0}/R=\Delta R/R\;$$

where $\Delta R\equiv R-R_{0}$ is the increment in the electrical resistance between the undamaged state and a generic damaged state. Equation (5) correctly gives the initial value $R=R_{0}$ when D=0, and R→∞ when D=1 (namely infinite resistance at the specimen rupture).

The time series of electrical resistance measurements, $r_{i}\left(t\right)\equiv R_{i}\left(t\right)/R_{0}$, is transformed into a series of energy events—manageable with the NT analysis method—making the following considerations:

- Each increase in electrical resistance is due to the creation of a new discontinuity surface in the conductive network of the specimen. According to Equation (5), each electrical resistance measurement $R_{i}$ is related to the effective current-conducting cross-sectional area $S_{i}$ of the cylindroid by:
  (6) $$R_{i}=\rho l/S_{i},\;i=0,\;1,\;2,\ldots$$
- The increase in electrical resistance $\Delta R_{1}\equiv R_{1}-R_{0}$, between the virgin state and the damaged state of the cylindroid, is related to the resulting surface of the freshly formed microcracks intersecting the cylindroid, expressed by $\Delta S_{1}\equiv S_{0}-S_{1}$. By exploiting Equation (6), it becomes:
  (7) $$\Delta S_{1}=(\rho l/R_{0})\left(1-R_{0}/R_{1}\right)=(\rho l/R_{0})\left(1-1/r_{1}\right)$$
- The subsequent increase $\Delta R_{2}\equiv R_{2}-R_{1}$ is related to the corresponding crack surface advancement $\Delta S_{2}\equiv S_{1}-S_{2}$ by:
  (8) $$\Delta S_{2}=(\rho l/R_{1})\left(1-R_{1}/R_{2}\right)=(\rho l/R_{0}r_{1})\left(1-r_{1}/r_{2}\right)$$
- At the generic step, $\Delta R_{i}\equiv R_{i}-R_{i-1}$ is related to $\Delta S_{i}\equiv S_{i-1}-S_{i}$ by:
  (9) $$\Delta S_{i}=(\rho l/R_{0}r_{i-1})\left(1-r_{i-1}/r_{i}\right)\;i=1,\;2,\ldots \;.r_{0}\equiv 1$$

If $G_{C}$ is the toughness of the material, the amount of energy dissipated over this cracking step is calculated by means of fracture mechanics:

(10) $$W_{i}=G_{C}\Delta S_{i}$$

- Therefore, the experimental time-varying electrical resistance values $r_{i}\equiv R_{i}/R_{0}$ are transformed into a time series of point-like energy events $W_{i}$, expressed as functions of $r_{i}$:
  (11) $$r_{i}\to \Delta S_{i}\propto W_{i}\propto \frac{1}{r_{i-1}}\left(1-\frac{r_{i-1}}{r_{i}}\right)$$

**Figure 4:**
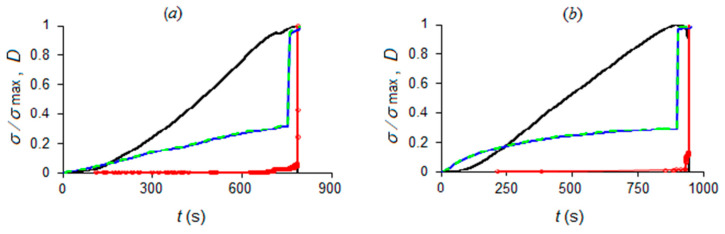

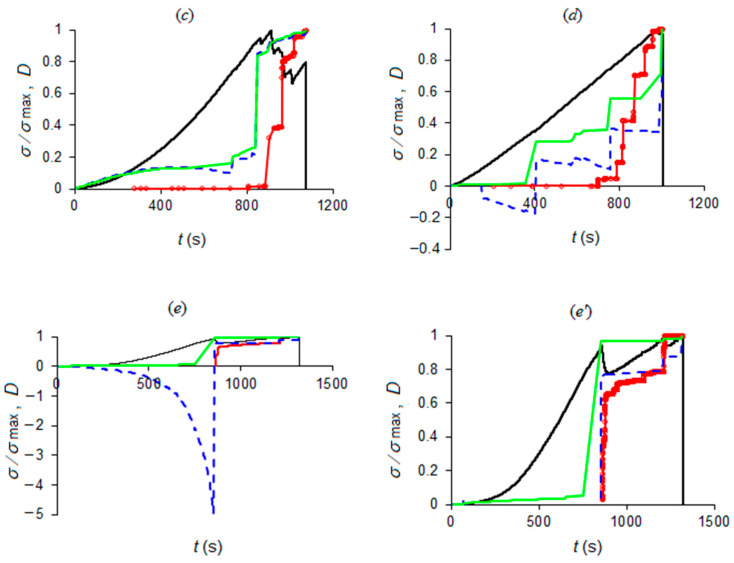
Normalized applied stress (black line) and damage evolution in terms of acoustic emissions (dotted red line); electrical resistance (dashed blue line); and crack surface advancements by indirect measurement (light green line). The diagrams refer to the specimens corresponding to the data presented in (**a**–**e**) (see inset table of Figure 3). The last picture (**e’**) is a detail of the positive half-plane (damage D>0): negative values are due to temporary decrements in the electrical resistance of Luserna specimen (**e**).

Trends of accumulated damage expressed by Equations (1) and (5) and by

(12) $$D\propto \sum_{i}\Delta S_{i}\;\propto \;\sum_{i}\frac{1}{r_{i-1}}\left(1-\frac{r_{i-1}}{r_{i}}\right)$$

(where Equation (11) is inserted) are depicted in Figure 4.

Due to the dependence of rock resistivity on the porosity (also known as Archie’s law [51], and here not explicitly considered), different trends in electrical resistivity were observed. The mortar specimens show a constant electrical resistance value up to the failure, whereas Luserna stone specimens are characterized by an initial decrease in the electrical resistance—presumably due to compaction caused by compressive loading—as emphasized by the negative part of the blue curves in Figure 4, representing damage in terms of electrical resistance according to Equation (5). They differ from the green curves, depicting damage in terms of freshly formed crack surfaces by Equation (12), where negative terms $\Delta R_{i}<0$ are discarded. Such contributions, i.e., those with $1-r_{i-1}/r_{i}<0$, are due to specimens’ compaction during the initial loading stages and are removed from the time series of the energy events, as being unrelated to the cracking process.

As it appears both in Figure 3 and Figure 4, while the mortar specimens are characterized by a significant electrical resistance variation only at the failure, significant electrical resistance changes, caused by internal cracking of the rock, were observed in Luserna stone in coincidence with stress drops.

Despite the fact that bursts of AE activity and significant changes in electrical resistance are clearly precursors of specimen failure, the critical point does not seem easily identifiable. The purpose of the following sections is to find the approach to criticality hidden in the specimens’ responses.

## 4. The Method of Natural Time Analysis

Although the NT method has been introduced for the analysis of SES (Seismic Electric Signals) and seismicity [52,53,54], it has been applied to a variety of signals providing optimal time-frequency enhancement [55]. The cornerstone of the NT analysis is the definition of a new time domain, the “natural time”, in which the “events”, i.e., the significant values of a time series, are equispaced, while their “energy” is retained, thus defining a new time series ($\chi_{k}$,$Q_{k}$), where $\chi_{k}=k/N$ is the NT, $Q_{k}$ is the energy of the k-th event, and N is the total number of successive events. In other words, the transformation of a time series from the conventional time to the NT focuses on the (normalized) order of occurrence of the events and ignores the time intervals between them [38].

According to the NT method, the approach to criticality is manifested by satisfaction of the following set of criteria: convergence of the parameter $\kappa_{1}=\sum_{k=1}^{N}p_{k}\chi_{k}^{2}-{\left(\sum_{k=1}^{N}p_{k}\chi_{k}\right)}^{2}$ to the value 0.070, while simultaneously the entropy in NT, $S_{nt}=\sum_{k=1}^{N}p_{k}\chi_{k}ln\chi_{k}-\left(\sum_{k=1}^{N}p_{k}\chi_{k}\right)ln\left(\sum_{k=1}^{N}p_{k}\chi_{k}\right)$, and the entropy under time-reversal, $S_{nt-}$, satisfy the condition $S_{nt},S_{nt-}<S_{u}=\left(\frac{\ln2}{2}\right)-\frac{1}{4}\left(\approx 0.0966\right)$, where $p_{k}=\frac{Q_{k}}{\sum_{n=1}^{N}Q_{n}}$ is the energy of the k-th event normalized by the total energy and $S_{u}$ denotes the entropy in NT of a “uniform” noise [56,57]. This set of criteria has been successfully applied to a variety of unprocessed EM signals which are possibly earthquake (EQ) related, such as the SES [57,58,59] and the MHz fracto-EME [8,9,60,61], to reveal the approach of the underlying dynamical system to criticality. Both for models of dynamical systems (such as the Ising model and several models of self-organized criticality) and real systems, the value $\kappa_{1}=0.070$ is being considered to quantify the extent of systems’ organization at the commencement of the critical stage [38].

Moreover, NT analysis is also applied to quantities, usually daily-valued ones, calculated from measured time series, such as recordings of ground-based magnetometers [56,62,63,64], and very low frequency (VLF) receiver recordings of sub-ionospheric propagation [65]. However, these quantities form time series of limited length (limited number of data), as happens with foreshock seismicity time series. In such cases, the set of criteria checked for the reveal of the approach to criticality is different and follows the paradigm of NT analysis of foreshock seismicity [52,54,57,66]. Finally, the identification of the approach to criticality in more complex systems calls for the investigation of the evolution of the entropy change ΔS ($=S_{nt}-S_{nt-}$) under time reversal [67,68], where the latter reference presents a methodology applicable (such as that in [69]) in specifically designed experiments to investigate AE activity in very long time series [68] or in different loading and unloading phases [69].

In more detail, for the case of foreshock seismicity, the evolution of specific NT analysis parameters versus time is studied by progressively including new events in the analysis until the time of occurrence of the main EQ event. These parameters are the already presented $\kappa_{1}$, $S_{nt}$ and $S_{nt-}$, as well as the “average” distance $\langle D\rangle =\langle |\Pi \left(\varpi \right)-\Pi_{critical}\left(\varpi \right)|\rangle$ (ϖ=2πφ, with φ standing for the frequency in NT) between the curves of normalized power spectra $\Pi \left(\varpi \right)={\left|\overset{N}{\underset{k=1}{\sum }}p_{k}exp\left(j\varpi \chi_{k}\right)\right|}^{2}$ of the evolving seismicity and the normalized power spectra at critical state calculated as $\Pi_{critical}\left(\varpi \right)\approx 1-\kappa_{1}\varpi^{2}$, for $\kappa_{1}=0.070$ [38]. Specifically, $\kappa_{1}$, $S_{nt}$, $S_{nt-}$ and 〈D〉 are re-calculated in each step, based on the rescaled time series ($\chi_{k}$,$Q_{k}$), as the total number N of the already included successive events is progressively increasing. In the resultant time evolution of $\kappa_{1}$, $S_{nt}$, $S_{nt-}$ and 〈D〉, criticality is considered to be truly achieved when, at the same time [38,59], (i) $\kappa_{1}$ approaches $\kappa_{1}=0.070$ “by descending from above”, (ii) $S_{nt},S_{nt-}<S_{u}$, (iii) $\langle D\rangle <10^{-2}$, and (iv) since the underlying process is expected to be self-similar, the time of criticality does not significantly change by varying the “magnitude” threshold.

In the application of seismicity NT analysis to other quantities, usually more than 20 threshold values equispaced between zero and 50% of the maximum value of the examined quantity are considered.

## 5. Analysis Results of Acoustic Emissions and Electrical Resistance Time Series

The application of NT analysis to AE acquired during laboratory experiments has already been addressed in [39], where NT analysis has been applied in direct analogy to the analysis of seismicity, as described in Section 4, while the quantity $Q_{k}$, the “energy” of each event, has been considered to be equal to the corresponding squared amplitude of each AE event, provided that this exceeds a certain threshold. In this work, for the case of the NT analysis of AE time series, we follow the same reasoning as [39]; specifically, we consider $Q_{k}=A_{\max,k}{}^{2}$, provided that this is higher than a certain threshold ${\left(A_{\max}{}^{2}\right)}_{Th}$. However, NT analysis of electrical resistance appears for the first time here. As shown in Section 3, the amount of energy dissipated over a cracking event, $W_{i}$, is directly related to the measured resistance values (see Equation (11)).

Although the quantity $Q_{k}$ in NT analysis corresponds to different physical quantities for various time series [38], an energy-related physical quantity is used for $Q_{k}$ where possible. Therefore, for the case of electrical resistance time series, we consider $Q_{k}=\left|W_{k}\right|$, provided that this is higher than a certain threshold $W_{Th}$, while NT analysis is also applied in direct analogy to the analysis of seismicity (Section 4).

**Figure 5:**
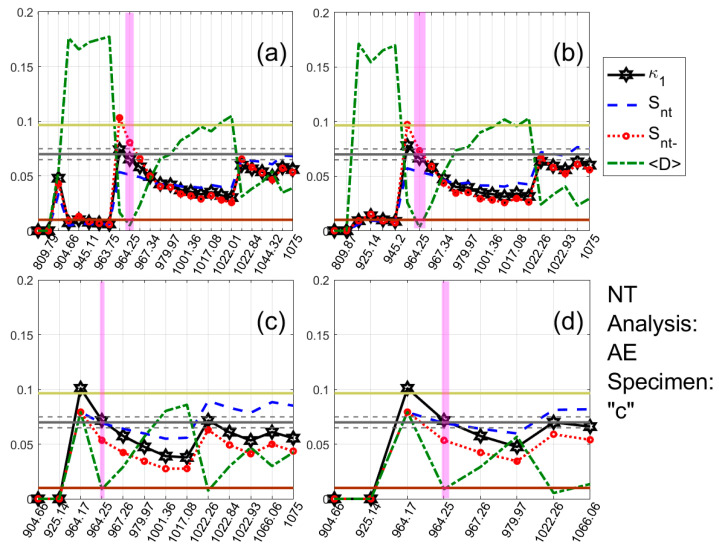
NT analysis of the AE time series acquired during the experiment involving specimen (*c*) of Figure 3. The different panels correspond to different threshold values ${\left(A_{\max}{}^{2}\right)}_{Th}$: (**a**) 0.1, (**b**) 0.2, (**c**) 0.7 and (**d**) 2. Each panel shows, on a common vertical axis, the variation of the values of all parameters of the applied NT analysis vs. time in seconds (0 s corresponds to the initiation of the experiment). The vertical magenta patches highlight the time when criticality is approached for each threshold, i.e., when criticality conditions (cf. Section 4) are satisfied. For the ease of interpreting the results, the $\kappa_{1}=0.070$ value is shown as a solid grey horizontal line, while the following limit values have also been depicted by horizontal lines: 〈D〉 limit ($10^{-2}$ ), solid brown line; entropy limit $S_{u}\left(\approx 0.0966\right)$, solid light green. The horizontal grey dashed lines at 0.070 ± 0.005 define the limits of the zone within which a calculated $\kappa_{1}$ value is considered to be ≈0.070. Note that the employed events are presented equispaced in the horizontal axis following the NT representation, but the time values presented are conventional time values for easier identification of the conventional time of approach to criticality; therefore, the horizontal axis is not linear in terms of the conventional time.

**Figure 6:**
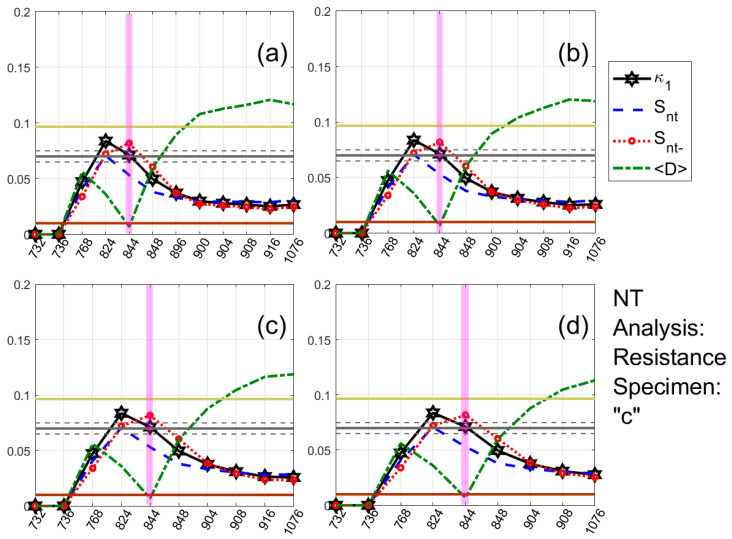
NT analysis of the electrical resistance time series acquired during the experiment involving specimen (*c*) of Figure 3. Variations of the NT analysis parameter values vs. time in seconds (0 s corresponds to the initiation of the experiment) for four different thresholds $W_{Th}$: (**a**) 0.00825, (**b**) 0.00975, (**c**) 0.01 and (**d**) 0.0105. The format of this figure follows the format of Figure 5.

One typical example of the results obtained for each type of the recorded time series (AE and electrical resistance) is presented in Figure 5 and Figure 6, respectively. These show the temporal evolution of the NT parameters $\kappa_{1}$, $S_{nt}$, $S_{nt-}$ and 〈D〉 for four threshold values of AE (Figure 5) and electrical resistance (Figure 6) of the Luserna stone specimen presented in Figure 3c and Figure 4c.

For both of them, the same NT analysis procedure has been followed: for each threshold value, the values of the time series under analysis (i.e., $A_{\max}{}^{2}\left(t\right)$ or |W(t)|, calculated from the original AE or electrical resistance time series, respectively, with t denoting the conventional time) are sequentially compared to the corresponding threshold (${\left(A_{\max}{}^{2}\right)}_{Th}$ or $W_{Th}$, respectively) and as soon as a value of the time series under analysis exceeds the threshold, a new event is included in the NT analysis, leading to a rescaling of the ($\chi_{k}$,$Q_{k}$) time series and recalculation of $\kappa_{1}$, $S_{nt}$, $S_{nt-}$ and 〈D〉. In Figure 5 and Figure 6, the magenta patches highlight when NT analysis criticality conditions are satisfied for each threshold value. As apparent from Figure 5 and Figure 6, during these highlighted periods, the criteria (i)–(iii) (see the application of NT analysis to seismicity in Section 4) for the approach to criticality are simultaneously satisfied, since $\kappa_{1}$ approaches the value $\kappa_{1}=0.070$ “by descending from above”, $S_{nt},S_{nt-}<S_{u}\left(\approx 0.0966\right)$, and $\langle D\rangle <10^{-2}$. For the AE case (Figure 5), the critical state is truly achieved in 964.25 s, the time that the highlighted periods for the presented four thresholds are overlapping, since, for this time, the criterion (iv) (see the application of NT analysis to seismicity in Section 4) is also satisfied. Correspondingly, for the electrical resistance case (Figure 6), the critical state is, according to the criterion (iv), truly achieved in 844 s, since, in that time, there is an overlap of the criticality periods for different thresholds.

**Figure 7:**
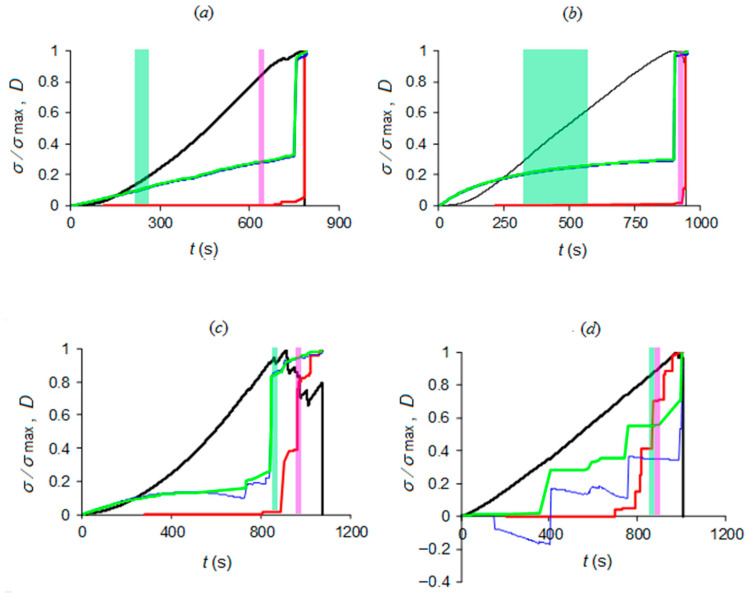

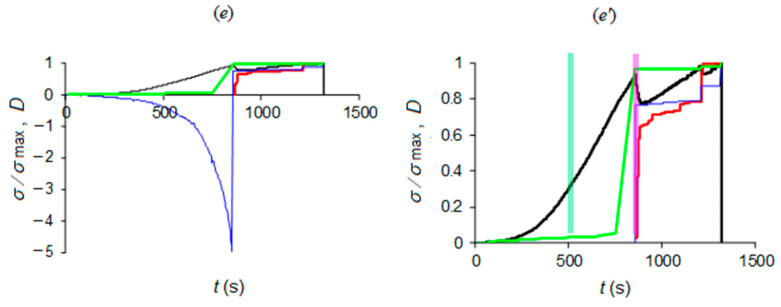
Times of approach to criticality by NT analysis (identified by magenta patch for AE and light green patch for electrical resistance) correlated to load history and damage evolution: the diagrams refer to CM specimens (**a**,**b**) and LS specimens (**c**–**e**) according to the inset table of Figure 3; last picture (**e’**) magnifies the positive damage range of (**e**).

By applying the same analysis procedure for the acquired AE and electrical resistance time series of all four specimens (Luserna stone and cement mortar; see Figure 3), the results presented in Table 2 were obtained. The obtained times of approach to criticality are also depicted in Figure 7 relative to the evolution of normalized applied stress and damage in terms of AE and electrical resistance.

As a general remark, criticality in terms of electrical resistance changes systematically precedes AE criticality for all investigated specimens (Table 2, Figure 7). This result could be somehow expected, since the recorded AE activity lies in a frequency range—from a few hertz to 10 kHz—related to large cracks. As a matter of fact, previous studies [45] showed that such low-frequency AEs take place later than high-frequency AEs due to microcracking and are to be regarded as late failure precursors.

Moreover, the position of the time of criticality relative to damage evolution (see Figure 7c), the two kinds of materials that can be summarized as follows:

(i) For cement mortar specimens, AE criticality appears close to the point where the damage evolution in terms of AE (red curves) starts to quickly rise, while electrical resistance criticality appears before damage evolution in terms of electrical resistance (blue curves) starts to quickly rise, at a time period where damage evolution in terms of electrical resistance has still a mild increase rate.

(ii) For Luserna stone specimens, AE criticality appears when the damage evolution in terms of AE (red curves) has already entered quick increase. For specimens (*c*) and (*d*), this occurs at or below 50% of the *y*-axis, during or just before the last-but-one “jump”, while for specimen (*e*), this occurs during the negative values section, at an earlier point of damage evolution. On the other hand, electrical resistance criticality appears before damage evolution in terms of electrical resistance (blue curves) and has already entered quick increase, above 50% of the *y*-axis, during or just before the last-but-one “jump”.

Then, some light is shed on a controversial result concerning Luserna stone, focusing on two specific examples, (*c*) and (*d*). In the case of Luserna specimen (*d*), the NT critical condition for the AE time series seems to be threshold-dependent, since criticality is reached at ~900.65 s for high threshold values and earlier, i.e., at ~790.32 s, for low threshold values. Such a threshold-dependent behavior can be interpreted as a deviation from self-similarity whereby a phenomenon, reproducing itself on different time, space and magnitude scales, is substantially threshold-independent. On the other hand, the uniqueness of the NT criticality condition for Luserna specimen (*c*)—964.25 s for all thresholds—shows evidence of self-similarity in the AE dynamics.

These observations seem to be confirmed by the Gutenberg–Richter (GR) power-law distribution of AE amplitudes, illustrating self-similarity: $N\left(\geq A\right)\propto A^{-b}$, where *N* is the number of AEs with peak amplitude greater than *A*. However, it is well known that the GR distribution has to be modified for larger events due to finite size effects, by introducing either an exponential cut-off or a second power-law with a larger *b*-value beyond a cross-over magnitude [70].

**Figure 8:**
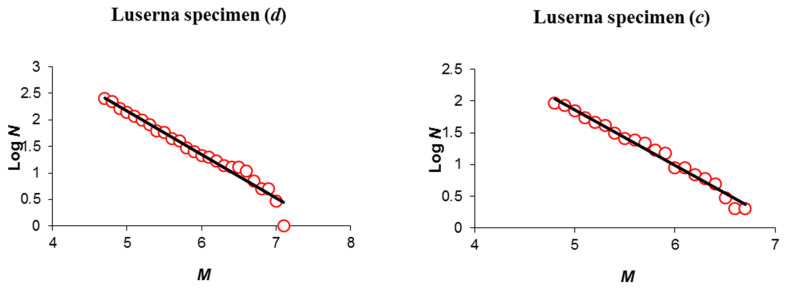
AE number *N* with magnitude greater than *M* as a function of *M* (*M* = log_10_ *A*) represented by red circles; the negative slope of the fitting line is the *b*-value. Deviations from linearity occurring at larger magnitudes (*M* ≥ 7) can be observed for the narrower specimen (***d***) (left panel), while the linear fit matches with experimental data of specimen (***c***) throughout the magnitude range (right panel).

As shown in Figure 8, the deviation from self-similarity at larger magnitudes is more pronounced in the GR distribution of specimen (*d*), whereby the reduced specimen diameter intervenes, introducing finite size effects.

**Figure 9:**
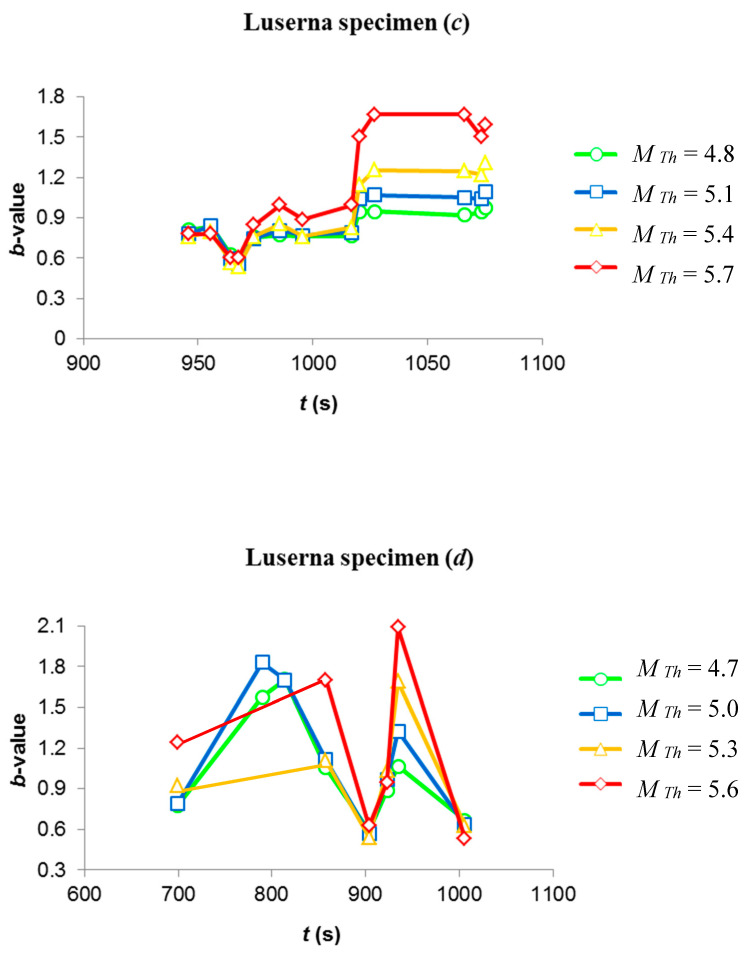
Trend of b-values for different threshold magnitudes. Trend lines of specimen (***c***) (upper panel) appear more self-similar than specimen (***d***) (lower panel).

Considerations about self-similarity could be done also in terms of the evolving *b*-values (Figure 9), where different trend lines are obtained using as many threshold values: for specimen *(c)*, trends are very similar and, then, threshold-independent, with a common minimum at 967.6 s. These findings are to be regarded as signatures of self-similar behavior. As regards specimen *(d)*, the trend lines of *b*-values exhibit a stronger dependence upon the threshold magnitude, with different positions of the minimum (903.91 s for *M_Th_* = 4.7, 5 and 5.3, and 1006.13 s for *M_Th_* = 5.6) reflecting the twofold NT criticality condition.

In both cases, it is observed an upward shift of the curves (i.e., larger *b*-values) by increasing the threshold, consistently with the “second power-law rule” of the modified GR distribution.

## 6. Conclusions

The present work focused on simultaneously acquired AE and electrical resistance time series, recorded during loading tests conducted on cement mortar (CM) and Luserna stone (LS) specimens.

It was observed a brittle mechanical response from CM specimens wherein, despite the absence of significant deviations from linear elasticity, the increase in electrical resistance and AE activity during the approach to failure revealed accumulation damage. On the contrary, except for the case (*d*), LS specimens were characterized by a more ductile behavior, with numerous intermediate load drops correlated to clusters of AE events and changes in electrical resistance.

These differences are reflected by the NT analysis of the recorded AE and electrical resistance time series, where electrical properties systematically reach criticality earlier than AE: the LS–AE criticality is reached in correspondence with significantly increased AE activity, and in correspondence with abrupt stress drops, whereas LS–electrical criticality can precede the major increments in the electrical resistance. The CM–AE criticality is reached at the onset of the damage rate acceleration before the specimen collapse—case (*b*)—or slightly earlier—case (*a*)—whereas the CM–electrical criticality is less easy to understand, as it is reached when the curve of the electrical resistance has not accelerated yet.

The apparently greater unpredictability of the CM fracture behavior could be ascribed to the different material properties: LS is a metamorphic rock with a heterogeneous structure, offering more abundant and more easily identifiable fracture precursors than an artificial material, compacted during the manufacturing process, and thus more homogeneous such as CM.

## Figures and Tables

**Table 1:** Chemical composition of mortar enriched with ferric oxide and Luserna stone. Weight percentage of silicon dioxide and ferric oxide added (mortar) is highlighted.

Mortar		Luserna Stone	
Element	% of Weight	Element	% of Weight
**SiO_2_**	**59.7**	**SiO_2_**	**72.0**
CaO	21.4	Al_2_O_3_	14.4
**Fe_2_O_3_**	**8.4**	K_2_O	4.1
Al_2_O_3_	3.3	Na_2_O	3.7
SO_3_	1.1	CaO	1.8
K_2_O	1.0	FeO	1.7
MgO	0.7	Fe_2_O_3_	1.2
Na_2_O	0.4	other oxides	1.1
other oxides	4.0		

**Table 2:** NT analysis results for the AE and electrical resistance time series of mortar enriched with iron oxide and Luserna stone specimens tested (cf. Figure 3).

Specimen (cf. Figure 3)	Material ^†^	Time of Approach to Criticality (s)	
		$\mathrm{NT}\;\mathrm{Analysis}\;\mathrm{of}\;\mathrm{AE}\;(A_{max,k}{}^{2})$	$\mathrm{NT}\;\mathrm{Analysis}\;\mathrm{of}\;\mathrm{Resistance}\;(\left|W_{k}\right|)$
(*a*)	CM	655–665	232–272
(*b*)	CM	934.83	328–580 ^††^
(*c*)	LS	964.25	844
(*d*)	LS	790.32 & 900.65 ^‡^	884 ^‡‡^
(*e*)	LS	866.54	508

^†^ CM: Cement mortar/LS: Luserna stone. ^††^ No approach to criticality was found for the dissipated energy (W) of specimen (b). However, the reported approach to criticality was identified after the NT analysis of power ($\frac{dW}{dt}$). ^‡^ NT analysis of specimen’s (d) AE yielded two times of approach to criticality: 790.32 s and 900.65 s, for low and high threshold, ${\left(A_{\max}{}^{2}\right)}_{Th}$, values, respectively. Note that the later approach to criticality (on 900.65 s) is more important as a possible precursor. ^‡‡^ The same time of approach to criticality was also identified after the NT analysis of power ($\frac{dW}{dt}$).
